# Implementing Goal-Directed Foraging Decisions of a Simpler Nervous System in Simulation

**DOI:** 10.1523/ENEURO.0400-17.2018

**Published:** 2018-03-01

**Authors:** Jeffrey W. Brown, Derek Caetano-Anollés, Marianne Catanho, Ekaterina Gribkova, Nathaniel Ryckman, Kun Tian, Mikhail Voloshin, Rhanor Gillette

**Affiliations:** 1 University of Illinois College of Medicine at Urbana-Champaign, University of Illinois at Urbana-Champaign, Urbana, IL 61801; 2 Max-Planck-Institut für Evolutionsbiologie, Plön D-24306, Germany; 3 Department of Bioengineering, University of California-San Diego, La Jolla, CA 92093-0412; 4 Neuroscience Program, Beckman Institute, University of Illinois at Urbana-Champaign, IL 61801; 5 University Library, University of Illinois at Urbana-Champaign, Urbana, IL 61801; 6 Department of Biology, Emory University, Atlanta, GA 30322; 7 Mighty Data, Inc, Brooklyn, NY 11225; 8 Department of Molecular and Integrative Physiology and the Beckman Institute for Advanced Science and Technology, University of Illinois at Urbana-Champaign, Urbana, IL 61801

**Keywords:** decision, homeostasis, learning, motivation, pleurobranchaea, simulation

## Abstract

Economic decisions arise from evaluation of alternative actions in contexts of motivation and memory. In the predatory sea-slug *Pleurobranchaea* the economic decisions of foraging are found to occur by the workings of a simple, affectively controlled homeostat with learning abilities. Here, the neuronal circuit relations for approach-avoidance choice of *Pleurobranchaea* are expressed and tested in the foraging simulation Cyberslug. Choice is organized around appetitive state as a moment-to-moment integration of sensation, motivation (satiation/hunger), and memory. Appetitive state controls a switch for approach vs. avoidance turn responses to sensation. Sensory stimuli are separately integrated for incentive value into appetitive state, and for prey location (stimulus place) into mapping motor response. Learning interacts with satiation to regulate prey choice affectively. The virtual predator realistically reproduces the decisions of the real one in varying circumstances and satisfies optimal foraging criteria. The basic relations are open to experimental embellishment toward enhanced neural and behavioral complexity in simulation, as was the ancestral bilaterian nervous system in evolution.

## Visual Abstract

**Figure:**
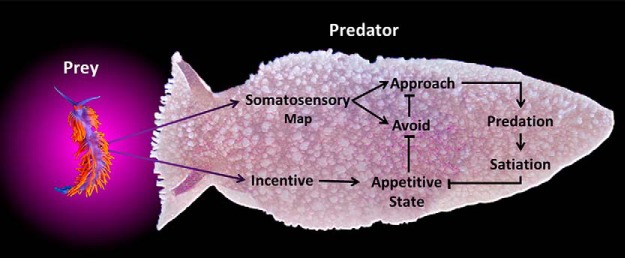

## Significance Statement

Contemporary artificial intelligence lacks the attributes of natural intelligence, in particular the abilities to relate information affectively. Accordingly, it is notable that the most complex animal behaviors serve primitive homeostatic goals, and emerge from the primitive mechanisms generating motivation and reward learning. Here is shown in simulation the function of a basic neuronal circuit for cost-benefit decision, derived from studies of a predatory generalist, the sea-slug *Pleurobranchaea*, and based on affective integration of information. Its simplicity may reflect distant ancestral qualities on which complexities in economic, cognitive, and social behaviors were built. The simulation validates experimental data and provides a basic module for expansion of behavioral complexity.

## Introduction

Foraging behavior in tracking and consuming resources is a series of economic decisions guided by stimulus characters predictive of risk and resource value. The basic behavioral choice is between an approach or avoidance of salient stimuli, a cost-benefit calculation done through integrating stimulus properties with motivation and memory. However, the natural intelligence displayed by even the simplest foraging animals has remained to be captured fully in artificial intelligence constructs in terms of actual neural computations made by real animal foragers.

We undertook to implement and test a model of foraging decision derived from the neuronal circuitry underlying approach-avoidance decisions in the predatory sea-slug *Pleurobranchaea*. The neuronal circuitry of decision has been characterized down to the single-neuron level (Gillette et al., 1982; London and Gillette, 1986; Jing and Gillette, 2000, 2003; Hirayama and Gillette, 2012; Hirayama et al., 2012, 2014). In particular, a key decision mechanism was found to lie in regulation of the turn motor network by the feeding network, whose excitation state depends on sensory input, memory, and satiation. Sufficient excitation in the feeding network converts default avoidance responses to sensory stimuli to approaching turns (Hirayama and Gillette, 2012). These findings localized motivation, appetitive state, and control of motor decision to the feeding network. They also account for behavior in which (1) quite hungry specimens not only orient to and bite at weak appetitive stimuli, but will also attack moderately noxious stimuli; (2) appetitive thresholds for approaching turns rise proportionately with satiation; and (3) as satiation increases, the animals avoid increasingly strong appetitive stimuli (Gillette et al., 2000; Noboa and Gillette 2013). Further, motor choice in *Pleurobranchaea*’s learned discrimination of odors paired with unconditioned stimuli (USs) is also mediated at the feeding motor network level (Davis et al., 1980; Mpitsos and Cohan, 1986a; Noboa and Gillette, 2013). These relations indicate a simple neural model for cost-benefit-based decision in the animal’s foraging (Gillette et al., 2000; Hirayama et al., 2012).

The simulation Cyberslug implements the integrated model in an autonomous agent with behavior designed from neurophysiological and behavioral data. To our knowledge no other such empirically driven neuroeconomic simulation has yet been devised. The success and utility of Cyberslug are supported through its accurate and dynamic reproduction of functional relations in *Pleurobranchaea*’s nervous system and behavioral repertory, its ability to maintain the fitness (here, nutritional state) of a virtual predator through plausible choices of differently valued prey based on hunger state, sensation, and memory; and its capacity to weigh risk against resource value to optimize foraging decisions.

## Materials and Methods

### Software accessibility

Cyberslug is freely available as extended data online at https://github.com/Entience/Cyberslug.

### General design of biological relations

Sigmoidal relations are used as constructive approximations to simulate biological processes that accelerate from small beginnings to saturate at high values. They or their influential values appear in Equations 3.0, 5.1, 6.0, 7.1, 7.2 to compute virtual place codes for sensory stimuli, appetitive stimulus affect, satiation, appetitive state, a behavioral switch based on appetitive state, and the amplitude of a turning response, respectively. Centers and asymptotes may be graphed for the interested reader from the values given in the source code.

### Learning

Reward and punishment associations are formed with the prey sensory signatures, *odor_hermi* and *odor_flab*, using the Rescorla-Wagner algorithm for classical conditioning (Rescorla and Wagner, 1972):

(1) ΔV=α∗β(λ−V).

On a given trial the change Δ*V* in the predictive value of a stimulus *V* (the amount of learning) depends on the difference between the value of what actually happens, *λ*, and what is expected (or already learned), *V*. The α term is the salience constant (the attention-getting capacity) of the conditioned stimulus (CS; ranging from 0 to 1, and set here at 0.5 for both *odor_hermi* and *odor_flab*). The β term is a rate parameter for the associative capacity of the US with the CS (ranging from 0 to 1; here a maximum of 1). The λ term is the maximum associative value of the US (set at 1 for *odor_hermi* and *odor_flab*). The Rescorla-Wagner algorithm was selected for its intuitive layout, simplicity, and robustness in a range of learning applications (Danks, 2003; Rasmussen et al., 2015). It is a useful approximation of learning from insects to mammals (Miller et al., 1995).

### Sensory transduction and integration

The Cyberslug agent uses bilaterally paired, anterior odor sensors, simplifying the real animal’s chemotactile oral veil’s function in prey tracking (Yafremava et al., 2007). The sensors report strengths of the three odors at slightly less than half a body length in front of the agent and at a roughly 40° angle with respect to its anteroposterior axis. For example, in the case of betaine, the averaged odor strength is

(2) $$sns\_betaine=\frac{sns\_betaine\_left+sns\_betaine\_right}{2},$$

where *sns_betaine_left* and *sns_betaine_right* are logarithmic functions of betaine virtual concentrations at each sensor. The *sns_betaine* variable integrates into the appetitive stimulus effect (Eq. 4.2).

The *Somatic_Map* function transforms sensory input into a virtual place code of the estimated direction of the strongest odor. It includes a mechanism emphasizing the salience of the nearest prey, analogous to surround suppression mechanisms underlying attention (Boehler et al., 2009): when closer to one prey type, sensation of the other is decreased, which reduces consumption of the aversively learned Flab in the presence of the odor of Hermi. The output of *Somatic_Map* is a template for the turn amplitudes of resulting approach-avoidance responses:

(3.1) $$Somatic\_Map=\left(\frac{sns\_flab\_left-sns\_flab\_right}{1+e^{-k0\;\cdot F}}+\frac{sns\_hermi\_left-sns\_hermi\_right}{1+e^{-k0\;\cdot H}}\right)$$

where

(3.2) F=sns_flab−sns_hermi

and

(3.3) H=sns_hermi−sns_flab.

The incentive variable (*Incentive*) integrates sensory information of positive and negative valences. It represents the incentive potential of a stimulus as modulated by learning and motivation:

(4.1) $$Incentive=R^{+}-R^{-},$$

where *R^+^* and *R^-^* represent appetitive and aversive stimulus affects, respectively:

(4.2) $$R^{+}=\frac{sns\_betaine}{1+k1\;\cdot \;V_{h}\;\cdot \;sns\_hermi}+k3\;\cdot \;V_{h}\;\cdot \;sns\_hermi$$

*R^+^* encodes the odor intensity of the primary resource indicator, betaine, in the first term. The positive association of Hermi odor becomes prominent with learning in the second term, and betaine values become less prominent. The variable *R^-^* represents the learned negative association of Flab odor. This variable might also encode negative effects of pain pathways, but in the present formulation it omits explicit pain and simply treats its consequences on aversive learning.

(4.3) $$R^{-}=k3\cdot V_{f}\cdot sns\_flab$$

### Satiation and appetitive state

Hunger state is represented through the function *Satiation*, which reflects *Nutrition* in a sigmoid relation with a lower bound near 0 and an upper asymptote at 1:

(5.1) $$Satiation=\frac{1}{{\left(1+k4\cdot e^{-4\;\cdot \;Nutrition+2}\right)}^{2}},$$

where *Nutrition*, without prey consumption, decreases recursively with each time step:

(5.2) $$Nutrition_{t+1}=Nutrition_{t}-0.0005\cdot Nutrition_{t}.$$

When feeding occurs, *Nutrition* is increased by a value of 0.3. The simulation initializes with *Nutrition* set at 0.8.

Appetitive state *(App_State)* is a function of *Incentive* and *Satiation*. It defines the thresholds for decisions to approach or avoid given prey items. It parallels expression of appetitive state in the excitation of *Pleurobranchaea*’s feeding network (Hirayama and Gillette, 2012). The sigmoidal element increases *App_State* as *Incentive* increases and decreases it as *Satiation* increases. As in the real animal, *App_State* is transiently suppressed during avoidance turning (J.W. Brown, V. Noboa, and R. Gillette, unpublished observations). This acts to further bias decision away from approach during the avoidance turn when some appetitive sensory input is present:

(6.0) $$App\_State=0.01+\frac{1}{\left(1+e^{-k5\;\cdot \;Incentive\;+\;k6\;\cdot \;Satiation}\right)}+k7\cdot (App\_State\_Switch-1),$$

where the expression (*App_State_Switch* – 1), defined and discussed later, causes a transient suppression of *App_State* during avoidance turns. It may be noted that satiation state is a variable in both *Incentive* and *App_State*, which reflects findings that satiation state is expressed in the basal excitation state of the feeding network, and that satiation may modulate sensory gain in the periphery (V. Noboa, T. Achler, and R. Gillette unpublished observations).

The satiation term dominates *App_State* values at its extreme ranges (0 and 1). Incentive is significant in the mid-range, as in *Pleurobranchaea* (Gillette et al., 2000). When satiation is either very low or high, it dominates over the incentive term. When very low, the Cyberslug agent chooses to consume the previously learned, noxious Flab. When very high, it actively avoids otherwise appetitive Hermis. These choices reproduce those made by very satiated or hungry *Pleurobranchaea* (Gillette et al., 2000; Noboa and Gillette, 2013).

### Turning and locomotion

The function *App_State_Switch* switches the turn motor network from avoidance to approach, representing the ASw_1,2_ actions of Figure 2. It acts like the corollary outputs from *Pleurobranchaea*’s feeding network that toggle the turn motor network polarity (Brown, 2014). The function converges steeply to either 1 or -1 depending on the value of *App_State*:

(7.1) $$App\_State\_Switch=\frac{-2}{1+e^{-k8\;\cdot \;\left(App\_State\;-\;0.245\right)}}+1,$$

In particular, when *App_State* goes below or above a threshold, set here as 0.245, *App_State_Switch* approaches 1 or -1, respectively, to govern turn direction. This threshold might well be a variable influenced by reproductive state, health, or neuromodulatory inputs from other neuronal networks (cf Hirayama et al., 2014), but is set as a simple constant here.

The turn is computed in degrees as:

(7.2) $$Turn\_Angle=\frac{k9\cdot App\_State\_Switch}{1+e^{3\;\cdot \;Somatic\_Map}}-App\_State\_Switch,$$

where positive or negative values of *App_State_Switch* cause avoidance or approach turns, respectively. Sufficient excitation in the Feeding Network (when *App_State* is >0.245) switches the polarity of an elicited turn from avoidance to approaching, while *Somatic_Map* determines turn magnitude by supplying somatotopic information on stimulus location.

When not actively engaged in prey approach or avoidance, the Cyberslug agent pursues a wandering trajectory, given as

(7.3) Turn_Angle=−1+random_float(2)

The *random_float* function generates a floating-point number between 0 and 2. In this case, it causes random changes in heading ranging from -1 to 1 degree on each time step.

## Results

### The core model

**Figure 1.**
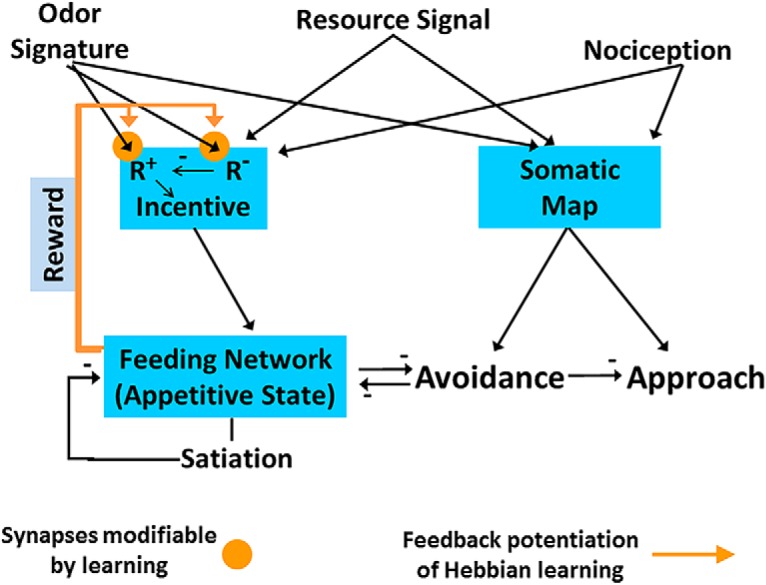
Approach-avoidance modeling in *Pleurobranchaea*. Appetitive state (excitation of the feeding network) summates intrinsic and learned stimulus values (incentive) with satiety to regulate turn response direction. In parallel, a somatotopic map of a stimulus in the animal’s oral veil sets the turn trajectory. Incentive sums sensory inputs predicting intrinsic nutritional value (resource signal) and the learned positive and negative values of prey odor signatures (R+ and R-). The positive or negative consequences of attacking the different prey are learned through instructive feedback from the feeding network. In the absence of incentive, basal appetitive state simply represents the animal’s satiation state (a negative feedback from prey capture). At some threshold, feeding network outputs change the turn motor response to a stimulus from default avoidance to an approach turn. A sensory place code (somatic map) for stimuli provides a template for turn response amplitude in both approach and avoidance. Negative feedback to the feeding network from the turn network during avoidance transiently suppresses feeding, while feeding network activity is reduced as satiation increases. The model is modified from Gillette et al., 2000; Hirayama et al. 2012.

**Figure 2.**
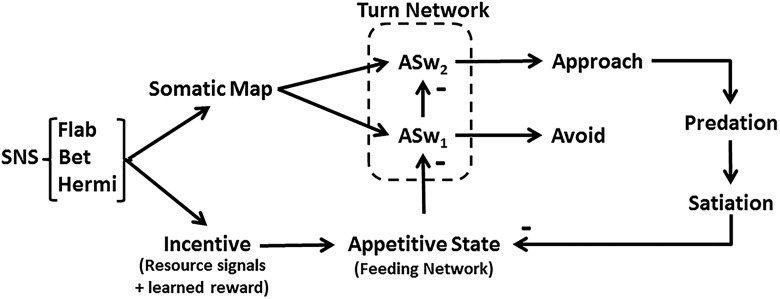
Logical flow in the Cyberslug model. Sensory inputs (SNS) for resource signal odor (Bet) and learned values of prey odor signatures (Flab and Hermi) are integrated into Incentive and summate with Satiation in appetitive state (App_State). Sensory somatotopic place information is encoded in somatic map, which acts as a template for the turn response amplitude. The turn motor network responds by default to sensory input with an avoidance turn response unless input from App_State is high enough to switch the turn to approach; this is mediated directly by a simple dyadic disinhibitory switch (ASw_1,2_). Successful predation increases satiation which in return reduces App_State.

Cyberslug is based on neuronal relations of approach-avoidance decision in *Pleurobranchaea*’s responses to odors of potential prey (Gillette et al., 2000; Hirayama and Gillette, 2012). Figure 1 shows the flow of information, from initial feature extraction of different sensory inputs to their evaluation in terms of estimated total resource value (incentive), based on nutritional need and memory; these processes then direct motor output for approach or avoidance turns. Figure 2 shows the logic of the model’s implementation into Cyberslug. The relations are represented in simple equations that drive the agent. Values of the constants (*K*_n_) in the equations were optimized over numerous trials and are found in the NetLogo code available as extended data. Altering the values in the code may lend appreciation for the role of natural selection in adaptively tuning neural circuitry.

Foraging decision is controlled by appetitive state. The animal’s feeding motor network is at the core of the decision module. Its excitation state directs choice between approach and avoidance turns (Hirayama and Gillette, 2012). The excitation state manifests the appetitive state of the animal; i.e., the disposition to engage in goal-directed appetitive behavior. Appetitive state integrates the animal’s satiation state, sensation, and memory of experience (Davis and Gillette, 1978; Davis et al., 1980; London and Gillette, 1986; Gillette et al., 2000; Hirayama and Gillette, 2012). Satiation determines the baseline excitation state of the feeding network. Incoming sensory inputs are integrated with memory into incentive. Incentive sums with satiation in the feeding network, either increasing or decreasing appetitive state.

By default, when appetitive state is low, the animal’s nervous system is organized so that the turn response to any sensory stimulus is avoidance. During the aversive turn appetitive state is decreased by inhibitory inputs in the feeding network (Davis and Gillette, 1978; London and Gillette, 1986; Hirayama and Gillette, 2012; Brown, 2014). Increasing appetitive state inverts the turn response direction to one of approach. Thus, appetitive state determines the sensory thresholds for the approach turn toward a prey and subsequent feeding responses. When high enough, corollary outputs from the feeding network appear to switch the excitatory sensory input-encoding stimulus site from one side of the turn network to the other, resulting in a turn toward the stimulus (Brown, 2014).

Sensory inputs here are of four kinds: (1) a resource odor signal predicting nutritional content to *Pleurobranchaea*, the amino acid betaine (Gillette et al., 2000); (2) a specific odor signature for a particular prey species (Noboa and Gillette, 2013); and (3) a place code for the averaged site of sensory input to the sensors (Yafremava et al., 2007; Yafremava and Gillette, 2011). (1) and (2) are summed as Incentive for resource and learned positive and negative values of prey odors (R^+^ and R^-^, respectively), which is then integrated with motivation (satiation) as appetitive state in the feeding network. The somatic map variable embeds (3) as a template for turn response amplitude. Positive or negative classical learning are assumed consequences of feedback from the feeding network operating in feeding or avoidance modes, respectively.

The Cyberslug simulation preserves the basic interactions of feeding and turn networks in the control of the turn by appetitive state and aversive suppression of the feeding network. Simplifications include: (1) for learning, explicit pain mechanisms are omitted in favor of arbitrary consequences; (2) exploratory locomotion is the default action in absence of active avoidance or approach; and (3) the switch mechanism for turn direction is rendered as a sigmoidal equation.

### Cyberslug environment

Cyberslug is implemented in the graphic modeling, agent-based programming language NetLogo 5.3.1. (Wilensky, 1999). Agent actions and odor diffusion are executed in discrete time steps by underlying code, where agents run commands in a turn-taking mechanism to simulate concurrence. At each time step the agent positions, orientations, speeds, and odor intensities in individual patches are updated with the variables that control them.

**Figure 3.**
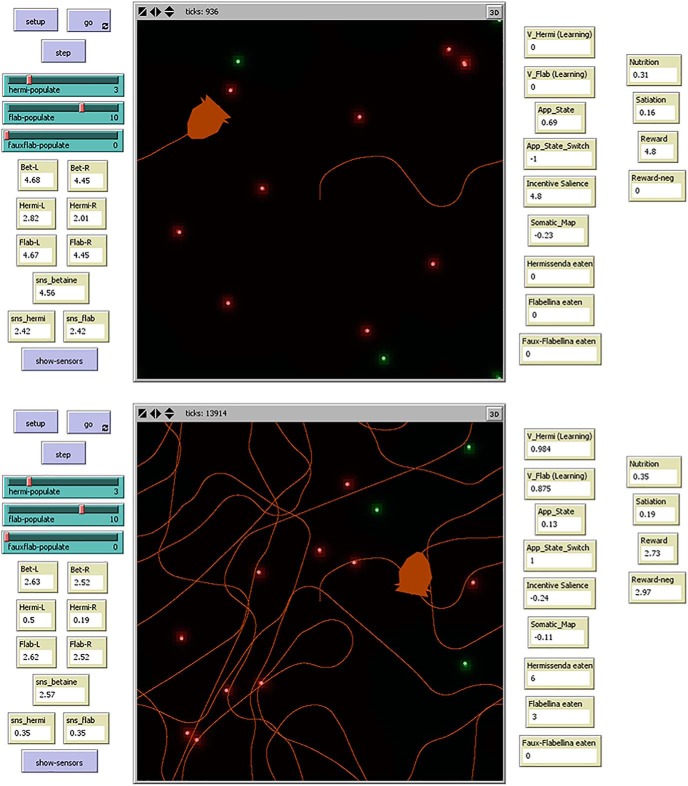
Screenshots of the Cyberslug environment and interface. Frames are shown from early (upper) and later (lower) in a software run. The Cyberslug agent (orange) encounters Hermi (green orbs) and Flab (red orbs) in its environment and traces its path (orange contours). Users can select the number of prey in the environment, move Cyberslug manually, or toggle the path tracer. Various Cyberslug and environmental parameters are updated in real time, as shown. In the early frame, the Cyberslug is orienting toward prey (App_State = 0.545, high; App_State_Switch = −1), and in the later frame, it is in aversive mode (App_State = 0.029, low; App_State_Switch = +1).

The interface screen (Fig. 3) displays current values of agent-associated variables and statistics. Users may override automatic agent navigation by manually controlling these agents with the mouse, specify the number of prey objects in the environment, and switch on or off a function that traces the agent’s path. When the simulation is initialized, a single Cyberslug agent and the different prey are generated at random positions in the environment.

### Prey

The Cyberslug agent encounters two virtual prey, “Hermi” and “Flab,” after the sea-slugs *Hermissenda crassicornis* and *Flabellina iodinea*, which *Pleurobranchaea* can encounter in the wild (Noboa and Gillette, 2013). These are shown as small orbs, colored green for Hermi and red for Flab. Each prey secretes two odors: the resource signal *betaine*, a predictor of nutritional resource (Gillette et al., 2000), and either of “*odor_hermi*” or “*odor_flab*.” Odors diffuse over time and space as for actual diffusion. Prey move in a simple random walk. Prey numbers remain constant; when consumed, replacements appear at random positions.

The specific odors of Hermi and Flab become associated with preference and avoidance, respectively, through reward learning. These effects are analogous to the ready consumption of *Hermissenda* by *Pleurobranchaea*, and the rejection and aversive learning of *Flabellina* (Noboa and Gillette, 2013). A Batesian mimic, “Faux-Flab,” is included as an option. In Nature, Batesian mimics receive protection from predation by mimicking appearance or odor of noxious species, and by their presence may increase attempted predation on the noxious species. Thus, Faux-Flab has the odor of Flab and the positive rewarding qualities of Hermi. The mimic is included for the user to test its effects on predator choices.

### The Cyberslug agent

Reward and punishment associations are formed with the prey sensory signatures using the Rescorla-Wagner algorithm for classical conditioning (Rescorla and Wagner, 1972), allowing the predator to learn through experience. Bilaterally paired, anterior odor sensors simplify the real animal’s chemotactile oral veil’s function in prey tracking (Yafremava and Gillette, 2011) to report the strengths of odors for betaine, a predictor of nutritional value, and the prey signature odors for *Hermissenda* and *Flabellina*. The sensors also transform sensory input into a virtual place code, giving the estimated direction of the source of the strongest odor, on which motor response is patterned. The incentive of an odor is calculated as summed positive and negative valences, which are determined by the intrinsic appetitive nature (for betaine) and learning experiences for the signature odors.

Appetitive state is the final regulator of behavioral choice. It summates incentive with satiation, and thus integrates sensory stimulus qualities with learning and motivation. Satiation is a simple function of nutritional state, which declines over time following prey consumption. Satiation dominates appetitive state at its extreme ranges (quite hungry or not); whereas stimulus incentive is significant in the mid-range. At a threshold value, appetitive state causes a directional switch between approach or avoidance in the turn response to an odor. The choices made reproduce those seen across the spectra of learning and hunger state by *Pleurobranchaea* (Gillette et al., 2000; Noboa and Gillette, 2013).

### Testing the simulation

Cyberslug was tested for prey selectivity as modified by learning, motivational state, and their interactions. Four sets of six tests were run under conditions assessing effects of learning and satiation mechanisms on prey selection. Effects of satiation and learning on selectivity were tested in arenas containing (1) 10 Flabs and 3 Hermis; (2) 13 Flabs alone; and (3) 13 Hermis alone. Tests ran for 150,000 software time steps.

**Figure 4.**
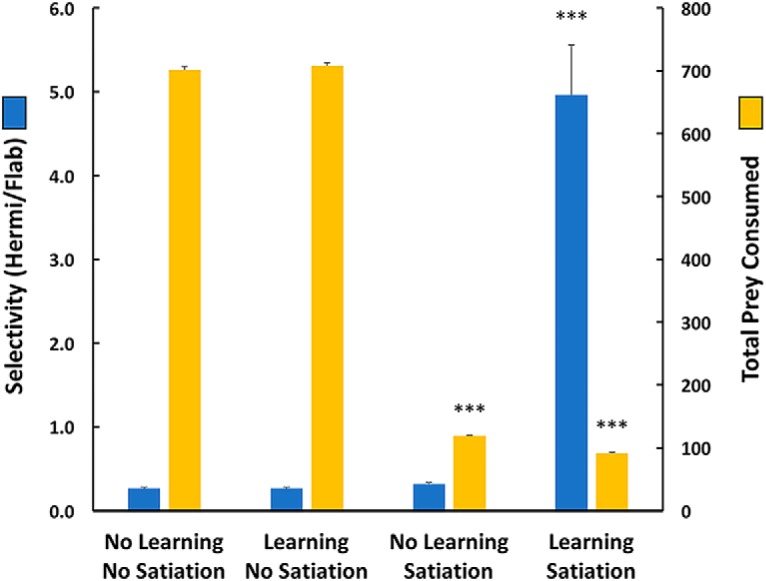
Effects of learning and satiation, and their interactions, on prey selectivity and total prey consumed. Without either mechanism for learning or satiation, selectivity was low, and prey were consumed as encountered in the 10 Flab/3 Hermi arena. Adding learning mechanisms did not alter either selectivity or number of prey consumed. When satiation was present without learning, prey consumed dropped, but selectivity was unchanged. When both learning and satiation mechanisms operated, selectivity was high and total prey consumed dropped to even lower values [one-way ANOVA across the four behavioral scenarios, *p* < 0.0001; ***p < 0.001 relative to all other learning/satiation scenarios (Tukey–Kramer, *n* = 6 trials in each)]. Differences in selectively between the first three learning/satiation scenarios, or in prey consumption where satiation was inactivated, were not significant (*p* > 0.05) Error bars are SEM. See text for further explanation.

Results in Figure 4 show that in the 10 Flab/3 Hermi arena satiation acted to limit the numbers of prey consumed between the two satiation and two no-satiation scenarios, while enhancement of prey selectivity depended on learning and satiation acting together. Thus, in tests where both learning and satiation mechanisms were inactivated, average total prey taken was 701 (SEM 6.22), of which 21.2% (SEM 0.6%) were Hermis, slightly less than their 23.1% frequency in the population. This yielded a selectivity value (total Hermis taken/total Flabs taken) of 0.27 (SEM 0.01), less than the 0.30 ratio of Hermis to Flabs in the population. This effect appeared due to a greater frequency of random clustering in the denser Flab population, leading to more frequent multiple consumptions of Flabs than Hermis. When learning was activated without satiation, average total prey taken was still high at 707.8 (SEM 5.5), of which 21.2% (SEM 0.6%) again were Hermis with still a low selectivity of 0.27 (SEM 0.01). Without learning, satiation alone reduced average prey consumed to 119 (SEM 0.52) with 24.1% (SEM 1.2%) Hermi, and with low selectivity of 0.32 (SEM 0.02).

Acting together, learning and satiation mechanisms led to an averaged total of 91.7 (1.43 SEM) prey taken, where 82.5% (SEM 1.5%) were Hermi. The selectivity coefficient of 4.96 (0.6 SEM) was a >18-fold increase over the values obtained without learning and/or satiation.

When tests were made in a field of 13 Flabs alone, with both learning and satiation intact, the averaged total prey taken was 53.3 (0.33 SEM). This low but nontrivial value reflected decisions to take noxious prey in a condition of extreme hunger, and also demonstrated combined effects of learning and satiation in reducing consumption of noxious prey (not shown). When similar runs were made in a field of 13 Hermis alone, the averaged total prey taken was 143.5 (0.81 SEM). The contrast of this value with the all Flab condition highlighted effects of positive versus negative learning in prey selection, as well as effects of satiation in limiting consumption. There were significant differences in number of prey consumed across all three arenas (*p* < 0.0001 in a one-way ANOVA; Tukey–Kramer, *p* < 0.001, among all three pairs).

## Discussion

The Cyberslug autonomous entity bases behavioral choice and perception on interactions of motivational state and learning, like the real animal. The algorithmic integration of sensation, motivational state, and memory reproduces adaptive action selection in behavioral choice. At intermediate values of satiation, the experienced Cyberslug agent selectively prefers or avoids the cues of benign or noxious prey, respectively. Otherwise, at lower levels of satiation (greater hunger) the predator is attracted to and consumes previously learned noxious prey. Accordingly, to forestall starvation it is economically realistic and in agreement with optimal foraging models that selectivity declines with decreasing satiation (Houston and McNamara, 1985). At higher satiation it actively avoids even the stronger appetitive signals. In these behaviors the simulation agrees with the classic, inverted U-shaped function relating arousal state to performance (Hebb, 1955), and reproduces major behavioral aspects of the real predator (Gillette et al., 2000; Noboa and Gillette, 2013).

The individual contributions of satiation and learning are naturally significant. However, the importance of their interactions in prey choice is well illustrated (Fig. 4). Without either one of satiation and learning, the unrestrained virtual predator takes in great quantities of either prey. This can be maladaptive to a real predator, where taking more high-quality prey than safely handled by digestion is physiologically threatening. Satiation limits the number of prey taken, but without learning noxious prey species are taken indiscriminately. Learning prey values promotes specific exploitation of the benign species and reduces attempts on the noxious species to periods of near-starvation, when a potential small benefit could be important to survival.

In Cyberslug, as in *Pleurobranchaea*, appetitive state is the continuous integration of sensation, internal state, and memory, and it sets the thresholds for expressing goal-directed behavior. Cyberslug summates the variables as appetitive state in the core Equation 6 to yield output that can switch avoidance responses to approach. Sensory integration in the model accomplishes two critical actions: evaluating the sensory stimuli as incentive, and providing a spatial map of stimulus location. Thus, incentive sums the primary odor nutritional signal (betaine) with positive and negative qualities learned from previous encounters with the initially neutral, specific odor signatures of its prey (Eq. 4.1).

In the absence of incentive input to the feeding network, appetitive state is solely dependent on the motivational variable satiation. However, with incentivized sensory input appetitive state becomes equivalent to “incentive salience” as defined in rodents and primates (Berridge and Robinson, 2016), where goal-oriented desire becomes tightly linked to reward cues and is critical to establishing preferences. The fuller concept of “motivational salience” regulating the attraction or aversion to objects in mammals (Puglisi-Allegra and Ventura, 2012) emerges in the present model with regulation of the approach-avoidance switch by appetitive state. Thus, the model illustrates how the salience of a stimulus may interact with motivational state and learning to determine its attractiveness or aversiveness.

Stimulus mapping is analogous to that done in the peripheral nervous system of the animal’s oral veil (Yafremava and Gillette, 2011): a virtual place code represents the averaged location of an odor stimulus as *Somatic_Map*, and incorporates an analog of lateral inhibition as seen in the animal (Eq. 3.1). This provides a template to map the motor output of the turn angle response, much like functions of superior colliculus and cortex in vertebrates.

Cyberslug implements essential elements of an affectively controlled, primitive type of immediate (or “anoetic,” unknowing) consciousness (Tulving, 1985) whose experience is largely a moment-to-moment event. It is a rudimentary form postulated as an evolutionary precursor to higher conscious functions of self-awareness in contexts of semantic and episodic memory (Tulving, 1985; Denton et al., 1999; Vandekerckhove and Panksepp, 2011; Vandekerckhove et al., 2014). In more complex animals, the simple immediate consciousness persists in the subpallial mechanisms that generate motivation and reward to drive homeostatic behavior, and which thereby sustain and direct the higher cognitive functions (Vandekerckhove and Panksepp, 2011). The rules governing choice in *Pleurobranchaea* and Cyberslug may resemble a core type of decision module present in ancestors of the major bilaterian lineages, before the evolution of the complex brains and behaviors that accompanied segmentation, articulated skeletons, and greater behavioral involvement in reproduction (Gillette and Brown, 2015). In the vertebrates, control of approach-avoidance decision is a basic function of the basal ganglia and hypothalamus. In arthropod nervous systems, these functions are performed by antennal lobes and mushroom bodies, which may conserve homologous structures as well as analogous functions (Strausfeld and Hirth, 2013). The feeding network in *Pleurobranchaea* combines functions of vertebrate hypothalamus and basal ganglia for motivation, incentive comparison, and selection of motor actions. The more complicated and modularized circuitries in vertebrates and arthropods reflect more complex bodies and lifestyles, but their brains were likely built onto a basic structure as shown here.

Little previous evidence has been found for empirically driven neuroeconomic simulations such as this one. However, it is notable that the innovative 1996 videogame Creatures used a bottom-up approach to AI character development, combining basic concepts of motivation and drive with Hebbian-like learning mechanisms in large artificial neural networks to achieve interesting behavior. This simulation is designed for transparency and interactiveness. Users may discover diverse, and perhaps unexpected, emergent properties for forager decision and prey vulnerability by altering their densities, particularly with the Batesian mimic, and by altering properties in the code.

The simple relations on which Cyberslug runs are readily adaptable to faster-executing computer languages, fine graphics, artificial neural networks, and neuromorphic representations. The present simple presentation of Cyberslug’s behavior in real time is intended to offer ready accessibility to a broad audience. The core decision module is open to practical improvements in learning algorithms, including addition of mechanisms for behavioral habituation and sensitization. More potential is present; for instance, the model embodies an essential character of the addictive process in incentivization, and might with little modification reproduce the sequelae of addiction, withdrawal, and cravings.

The homeostatic circuit relations underlying hunger drive in Cyberslug and *Pleurobranchaea* are adaptable to acquiring other resource types, such as hydration, salt balance, shelter, play, and social interactions, to name a notable few common to vertebrates. Truly intelligent and sentient virtual entities, defined in terms of empathic communication and abstract thought, may not yet exist because they lack the constellation of autonomy, motivation, valuation, emotion, and social awareness (cf. also Minsky, 2006). Of these, Cyberslug supplies essential aspects of autonomy, biologically based motivation, and valence assignment. It is reasonable that cognitive and social features might be added in simple piecemeal fashion following an evolutionarily plausible course, guided by comparative reference to invertebrate and vertebrate species that vary incrementally in their cognitive and social expressions with complexity of lifestyle. Of necessity in evolution, most valuation and decision processes in the economies of complex social animals would have been elaborated onto pre-existing, simpler decision modules for homeostasis, like those of *Pleurobranchaea* and other simple invertebrate foragers. The present relations are similarly open to embellishment in simulation.

## Synthesis

Reviewing Editor: Elizabeth Cropper, Mt Sinai Sch Med Decisions are customarily a result of the Reviewing Editor and the peer reviewers coming together and discussing their recommendations until a consensus is reached. When revisions are invited, a fact-based synthesis statement explaining their decision and outlining what is needed to prepare a revision will be listed below. The following reviewer(s) agreed to reveal their identity: Donald Edwards, Robert Huber

Reviewer #1

Understanding the operation of complex nervous systems to control behavior requires the creation of computational models that render the individual parts transparent as they work within the whole. The authors presented a model of nervous function and hunting/prey avoidance behavior of the marine slug Pleurobranchaea. They have abstracted mechanisms for satiation, behavioral incentive, odor tracking, approach/avoidance, and predation and combined them in a dynamic model of the sea slug as it operates in a virtual 2-D environment. There it pursues and consumes or avoids different prey items while learning which are ‘good’ (preferable to eat) and which are ‘bad’ (to avoid). The hunting behavior is conditioned by the degree of satiation, so that when less satiated, Cyberslug will more readily pursue the bad prey and when more satiated it will avoid them.

The reason for creating and testing the model was to show that such an autonomously behaving agent could be created, given that no such ‘empirically driven neuroeconomic simulation has yet been devised’. The model ‘works’ in that both learning and satiation control the model's behavior in a sensible way, causing it to be more discriminatory when sated and less discriminatory when hungry. This is a worthwhile accomplishment, but it begs the question of whether there is something more to be learned from the model: what predictions does it make about the animal's behavior or neural mechanisms, or what unexplained behaviors or circuitry does it account for? The initially surprising (to me) result was that learning appeared to have no effect on prey selectivity in the absence of satiation (left two bar plots in Fig. 4). However, if ‘no Satiation’ means that the Appetitive State is always high and learning is unable to affect it, then of course the model will be locked into an undiscriminating Approach mode. This seems to be more of an artifact of the model than a result that provides useful insights into the animal's behavioral mechanisms.

Models are interesting insofar as they extend our understanding. This model might be made more interesting by answering one or more questions: How does Cyberslug's performance depend on the choice of parameter values? How might the model be extended to predict or account for other phenomena? Are there model results that predict the outcome of interesting experiments?

Reviewer #2

The paper might consider including a brief section on the timing mechanism of netlogo and how it is implemented in CyberSlug. Also a discussion to what degree states of behavior, emerging from an analog, parallel processing system, can be modeled and approximated with a digital, discreet, serial processing framework.

Page 9, Line 198. “This threshold

might well be a variable influenced by reproductive state, health, or inputs from other neuronal

networks, but is set as a simple constant here.” A mention of neuromodulators, such as various amines and peptides, could be included here, they may well be the most prominent source of behavioral variation in this context.

In summary the paper introduces a very useful tool for exploring behavioral decisions, it implements rules, parameters, and states that are informed by knowledge of neural functioning for a set of its constituent elements. The graphical approach for monitoring changes in behavior allows the user to explore multiple scenarios and develop an understanding of the sensitivity of the system to different inputs.

## Author Response

Responses for Reviewer 1:

1) “...a worthwhile accomplishment, but it begs the question of whether there is something more to be learned from the model: what predictions does it make about the animal's behavior or neural mechanisms, or what unexplained behaviors or circuitry does it account for?”

What is to be learned from the model? Great point. Users will find unexpected effects of altering prey densities in various situations. We have made this simulation completely transparent and broadly accessible, which we think is unusual in most scientific computational modeling. In the model, under the ‘info’ button we suggest a variety of such experiments. For instance, it will be interesting for a user to find that with the Batesian Mimic there are rather narrow ranges where protection is given to the mimic in the densities and ratios of noxious species, mimics, and predators. We now refer in Discussion to emergent properties discoverable by interested users (Lines 470-473): “This simulation is designed for transparency and interactiveness. Users may discover diverse, and perhaps unexpected, emergent properties for forager decision and prey vulnerability by altering their densities, particularly with the Batesian mimic, and by altering properties in the code.”

2) “The...surprising...result was that learning appeared to have no effect on prey selectivity in the absence of satiation.”

We now point out in Discussion (lines 409-411), with a new reference, that previous models support the idea that indiscriminate choice can be adaptive, and it is economically realistic to many foragers for selectivity to decline with decreasing satiation. It will be explicitly demonstrated to the attentive user. We might expect animal species to vary widely in the effects of motivation on choice, depending on diverse ecological factors. Interested users can vary the motivational functions in the code to either limit or enhance its effects.

3) “How does Cyberslug's performance depend on the choice of parameter values?”

The choice of parameter values for Cyberslug is as critical as for real animals. We now refer to this in Methods. Cyberslug's were selected by its designers to optimize behavior with respect to its own sensory apparatus and environment (prey features). Whereas, for real animals the tuning is done analogously by natural selection. Workable parameters for Cyberslug might have been arrived at using genetic algorithms, but at this initial stage of its development it might have required a lot more work. The interested user can experiment with changing the values in the program. Lines 231-234 now state: “Values of the constants (Kn) in the equations were optimized over numerous trials and are found in the NetLogo code available as extended data. Altering the values in the code may lend appreciation of the role of natural selection in adaptively tuning neural circuitry.”

4) “How might the model be extended to predict or account for other phenomena? Are there model results that predict the outcome of interesting experiments?”

Are there model results that predict the outcome of interesting experiments? This also relates to our Response (1), above. We have added an optional system for experimental users in the Batesian Mimic, which is well described in the Cyberslug program ‘Info’ tab. For us, it was interesting to see the effects of varying the densities of the three different species in the protection of the mimic and loss of it in the true aposematic. We did not expect the interesting effects beforehand, and found later that they are documented and predicted in the ecological literature.

How to extend the model? We now briefly refer to a current direction in modeling addiction as it emerges from the simple foraging relations (lines 478-480): “More potential is present; for instance, the model embodies an essential character of the addictive process in incentivization, and might with little modification reproduce the sequelae of addiction, withdrawal, and cravings.”

Responses for Reviewer 2:

1) “The paper might consider including a brief section on the timing mechanism of netlogo and how it is implemented in CyberSlug. Also a discussion to what degree states of behavior, emerging from an analog, parallel processing system, can be modeled and approximated with a digital, discreet, serial processing framework.”

We appreciate the debates on serial vs. parallel processing of analog phenomena in computational neuroscience. With respect, we think that it would be digressing too far from the central points of the work to enter that discussion, as it would require more space than warranted here to address views on discrete simulation of continuous phenomena in serial processing. However, to hopefully clarify the software workings, we have modified the statement in Methods, lines 298-300, to read “Agent actions and odor diffusion are executed in discrete time steps by underlying code, where agents run commands in a turn-taking mechanism to simulate concurrence.” Those on either side of the debate can take away what they will.

2) “A mention of neuromodulators, such as various amines and peptides, could be included here, they may well be the most prominent source of behavioral variation in this context.”

Good idea. We have added the descriptor ‘neuromodulatory’ to the sentence and a reference to our paper in which effects of neuromodulation, both extrinsic and intrinsic, on behavioral thresholds are shown (lines 200-203): “This threshold might well be a variable influenced by reproductive state, health, or neuromodulatory inputs from other neuronal networks (cf. Hirayama and Gillette, 2012), but is set as a simple constant here.”
